# Performance Evaluation of Fan-Ventilated Swine Trailer with Air Filtration for Maintaining Satisfactory Transport Conditions

**DOI:** 10.3390/ani16010083

**Published:** 2025-12-27

**Authors:** Alvin Alvarado, Marjorette Baguindoc, Roger Bolo, Shelley Kirychuk, Bernardo Predicala

**Affiliations:** 1 Chemical and Biological Engineering, University of Saskatchewan, 57 Campus Dr., Saskatoon, SK S7N 5A9, Canada; alvin.alvarado@usask.ca (A.A.); mhb011@usask.ca (M.B.); reb908@usask.ca (R.B.); 2 Prairie Swine Centre Inc., 2105—8th St. East, P.O. Box 21057, Saskatoon, SK S7H 5N9, Canada; 3 Department of Medicine, University of Saskatchewan, 107 Wiggins Rd., Saskatoon, SK S7N 5E5, Canada; shelley.kirychuk@usask.ca

**Keywords:** animal transport, animal welfare, environmental condition, ventilation

## Abstract

In response to new and emerging challenges in animal transport, including more stringent biosecurity and public demand for enhanced animal welfare, an innovative prototype trailer with mechanical ventilation and air filtration systems was developed. The performance of the trailer in maintaining acceptable environmental conditions for the pigs during transport in both cold and warm weather was evaluated through a series of road tests. In these tests, the general welfare of the animals during transport was also assessed. Results showed that temperatures inside the animal compartment during cold ambient conditions were above 10 °C for more than 60 to 90% of the trip despite the frequent occurrence of cold temperatures (below 0 °C) at the inlet. On the other hand, the temperature in the animal compartment ranged from 16 to 19.4 °C most of the time during transport in warm weather. The average moisture levels in the animal compartment ranged from 4.15 to 6.3 g/kg dry air and 5.05 to 78.8 g/kg dry air during cold and warm transport conditions, respectively, which is comparable to the humidity ratios measured in conventional pig transport trailers. Carbon dioxide concentration inside the animal compartment ranged from 912 to 1192 ppm in cold conditions and from 1008 to 1414 ppm in warm weather, indicating good air quality in the trailer during transport. Furthermore, there was no significant change in the levels of blood cortisol and in the rectal and body temperatures of pigs measured at the start and end of each monitoring trip, indicating that the pigs showed reduced or minimal stress during transport. The study demonstrated that the trailer design with a mechanical ventilation system significantly improved the thermal comfort and environmental conditions for pigs, contributing to their welfare during transport.

## Simple Summary

This study addresses the growing concerns that current swine transport trailers expose animals to thermal stress, injury, and handling difficulties, thereby compromising animal welfare and contributing to significant economic losses within the Canadian swine industry. The primary goal of this study was to evaluate the performance of an enhanced swine trailer fitted with mechanical ventilation and air filtration systems, providing an acceptable environment during transport. Road tests with pigs were conducted in both cold (−10 to 10 °C) and warm (10 to 15 °C) weather to assess whether the installed ancillary systems and added trailer features could maintain required temperatures and air quality. Animal behavior, physiological stress indicators, and overall well-being were also monitored during transport. Results demonstrated that the enhanced trailer maintained more stable environmental conditions, reducing exposure to extreme temperatures and improving air quality relative to conventional swine transport trailers. Pigs (weighing 25 to 30 kg) transported in the enhanced trailer exhibited behavioral and physiological indicators of reduced stress and improved comfort. No injuries and transport-related morbidity and mortality were recorded during transport. These findings suggest that the adoption of enhanced trailer design can substantially improve animal welfare during transport while also reducing production losses and enhancing the economic sustainability of swine operations.

## 1. Introduction

Public demand for enhanced animal welfare in food animal production has increased significantly, partly due to recent high-profile cases concerning animal welfare during transport [1,2,3]. Animal welfare concerns during transport include the possibility of animals experiencing stress, injury, fatigue, mortality, and morbidity [1]. These issues are due to varying extreme environmental conditions inside the trailer, stress brought by multiple and steep internal ramps, difficulties during animal handling, and exposure to vibrations and noise, all of which are influenced by trailer design [4,5,6,7,8,9,10,11]. Current designs of animal transport trailers face significant challenges in ensuring both the welfare of animals and the economic viability of transport. A 4-year study conducted from 2012 to 2015 on the economic impact of transport losses in market-weight pigs in the US revealed a total loss of 0.88%, consisting of 0.26% losses due to DOA and 0.63% due to non-ambulatory pigs. This translates to annual losses of USD 89 million (USD 0.83 per pig marketed), with USD 52 million (USD 0.48 per pig marketed) attributed to DOA and USD 37 million (USD 0.35 per pig marketed) to non-ambulatory pigs [12].

Current trailer designs used in the commercial transport of pigs in North America vary from a small single-deck trailer to large three-deck drop center trailers (also known as potbelly). The thermal micro-environment within the transport vehicle poses the greatest risk to the animals’ welfare and well-being. A study conducted by Brown et al. [6] found that the lower front compartment of potbelly trailers was 10 °C warmer than the ambient outdoor temperature, which raised concern, especially during hot periods when outdoor temperature exceeds the critical temperature of 26 to 31 °C. Pot belly vehicles also feature multiple steep internal ramps (with a 40-degree slope) and sharp 180-degree turns, which complicate handling during loading and unloading. This design leads to increased use of electric prods and longer loading and unloading times [4]. In addition, negative effects on meat quality (i.e., pH, change in water-holding capacity, color defect) from transportation-related stress using conventional trailers have been reported [5,6,12,13].

Having identified the common causes of transport losses, particularly those associated with the design of the transport trailer, the swine industry is keen on finding solutions to provide the animals with a satisfactory environment during transport. Previous studies suggested re-designing the trailer and applying interventions on the existing livestock transport to reduce the impact on animal welfare, maintain pork quality, and avoid economic losses. Remedies to improve animal transport conditions include adding fans in critical locations [14], increasing side openings [15], using water sprinkling for evaporative cooling [12,16], and using effective boarding and bedding levels during mild and cold weather periods [17,18]. However, all of these past studies were conducted on naturally (passive) ventilated trailers, with limited research on the potential benefits of adding mechanical ventilation systems to livestock trailers to improve overall thermal condition and animal welfare during transport. To address this gap, this study developed a new prototype trailer fitted with air filtration and a ventilation system, along with enhanced structural features, designed to better protect animal welfare and maintain a reduced-pathogen environment inside the trailer during transport.

After completion of the enhanced prototype trailer, road tests with animals loaded in the developed trailer were necessary to properly assess the actual performance of the systems, with the pigs contributing latent and sensible heat and moisture in the trailer airspace, as well as to evaluate the overall welfare of the pigs when transported in this type of trailer. The goals of the road tests were to evaluate (1) the performance of the installed ancillary systems (ventilation and heating systems) in maintaining the optimum thermal and air quality environment for the pigs; (2) the general welfare of the animals during transport.

## 2. Materials and Methods

### 2.1. Description of the Prototype Trailer

**Figure 1:**
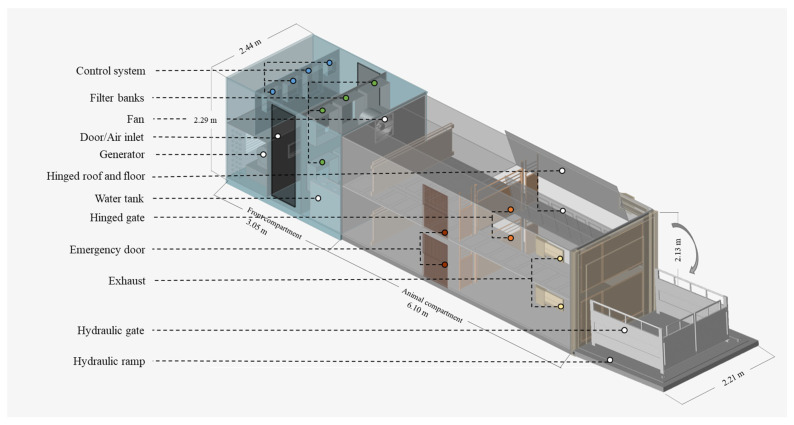
Diagram of the trailer compartment and components.

**Figure 2:**
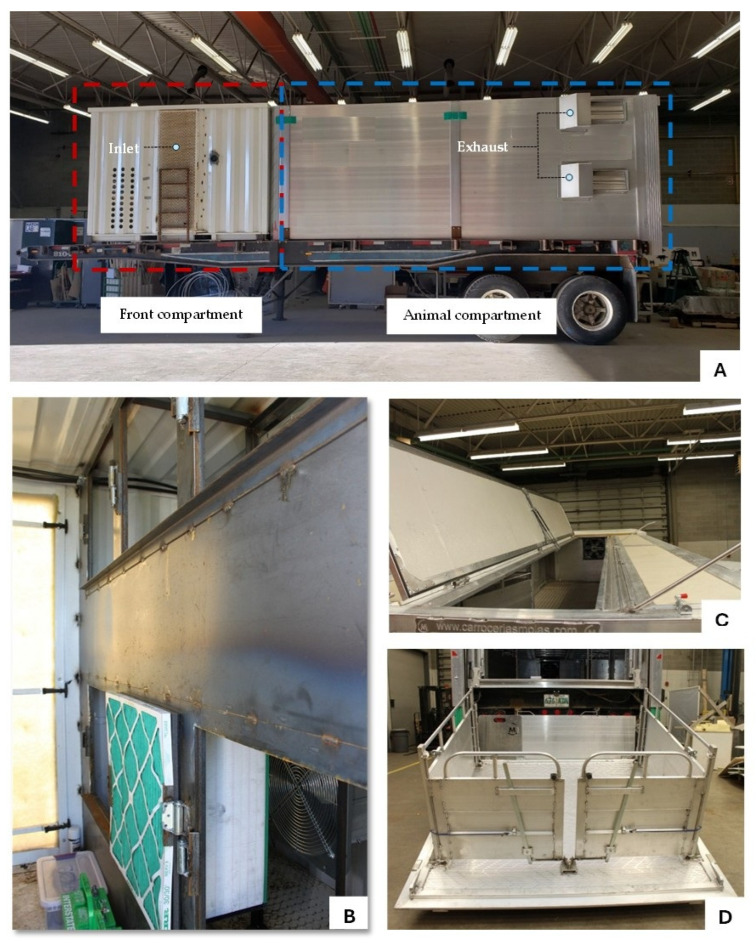
Photos of the prototype trailer showing the exterior of the front and animal compartment as well as the inlet and exhaust (**A**), filter banks inside the front compartment (**B**), hinged roof (**C**) and hydraulic lift gate (**D**).

The prototype trailer consisted of two main compartments: the front compartment and the animal compartment, both installed on top of a flatbed trailer (Figure 1 and Figure 2). It is a dual (top and bottom) straight-deck trailer, with totally enclosed, positive-pressure fan-ventilated animal compartments. The front compartment measured 3.05 m × 2.44 m × 2.29 m (l × w × h) and housed the ventilation and heating system components, drinking water and misting system, a 10-kW single phase generator set (PowerLineTM Model KS1000-T4, Frontier Power Products, Calgary, AB, Canada) to power the components in the trailer during transport, and six filter banks with each bank consisting of a 70 cm × 70 cm × 2.54 cm MERV 8 pre-filter (30/30, Camfil Farr, Calgary, AB, Canada) in series with a 70 cm × 70 cm × 30.48 cm MERV 16 main filter (Durafil ES, Calgary, Camfil Farr, AB, Canada). Three 900 W electric heaters were installed at the rear grill of the axial fans to heat the air supplied to the animal compartment to the desired temperature. In addition, an enhanced environmental control system with an independent control for the top and bottom deck fans was installed. The environmental control system was regulated by temperature, relative humidity (RH), and carbon dioxide (CO_2_) levels inside the trailer and has real-time datalogging features; the control system can be accessed while in transit as well as monitored and adjusted by the truck driver as needed.

The animal compartment measured 6.10 m × 2.21 m × 2.13 m (l × w × h) and had two identical decks (top and bottom). Each deck was divided into two pens (front and rear) by a hinged gate. Inlet air must pass through the filter bank in the front compartment before entering the animal compartment. Various features, such as a hydraulic loading platform (avoiding the need for ramps) and a hinged floor and roof section to facilitate loading/unloading and cleaning, were incorporated into the trailer design. In addition, sensors for temperature and RH (OM EL-USB-2 data loggers, Omega Engineering, Laval, QC, Canada; accuracy: ±0.5% (temperature) and ±3% (relative humidity)), CO_2_ (SE-0018 sensors, CO2Meter, Ormond Beach, FL, USA; range: 0 to 10,000 ppm; accuracy: ±3%), and air speed (D6F-W10A1 airspeed sensors, Omron, Shimogyo-ku, Kyoto, Japan; accuracy: ±5%) were installed inside the animal compartment and connected to an environmental controller (Zelio Logic SR2 Controller, Schneider Electric, Winnipeg, MB, Canada). All these features were integrated into the design to address issues identified in current conventional trailers used in the industry concerning animal welfare, biosecurity, cleanability, and lack of environmental control. Flooring was corrugated steel for better traction. Exhaust air dampers (Kehfab, Steinbach, MB, Canada) were installed at the rear compartment to prevent backflow of unfiltered air into the trailer. Each pen had one feeder. Feeders as well as the drinking water and misting system were not activated during transport; however, feed and water were kept readily available in case of an emergency or equipment malfunction that might require the pigs to remain in the trailer longer than expected. In addition to closed-circuit video cameras installed in both decks for real-time surveillance of pig activities inside the trailer, a multi-purpose access door with inspection hatches was installed on each deck. These allowed visual inspection of the pigs as well as pen equipment and sensors without compromising the biosecurity of the animals. Access doors also served as emergency unloading and ventilation openings in case of hydraulic lift and ventilation system malfunction.

### 2.2. Experimental Procedure

Nursery pigs weighing between 25 and 30 kg were loaded in the bottom deck compartment of the trailer and transported for 6–8 h during cold (−10 to 10 °C) and warm (−10 to 15 °C) weather conditions, covering a total distance of 600–800 km. A total of 16 monitoring trips were completed: twelve monitoring trips with 10 pigs per trip and four monitoring trips with 40 pigs per trip were conducted. The study was approved by the Animal Care and Ethics Board of the University of Saskatchewan (Animal Use Protocol number 20210078).

Based on the recommended stocking density of 0.15 m^2^/25-kg pig [19] and the floor space in each compartment, the maximum number of animals at this size that the trailer could hold was 80 pigs. However, to reduce the total number of experimental pigs per trip, which was an important consideration for Animal Care and Ethics approval of the study, only 10 pigs were loaded in the front section of the bottom deck of the trailer for each of the 12 road tests conducted. This configuration represented a condition in which the trailer was loaded only at partial capacity. A partition was installed near the fans to reduce the total pen area and maintain the recommended space allowance of 0.15 m^2^ per pig [19]. In addition, four road tests were conducted with a total of 40 pigs (20 pigs × 2 pens) loaded at the bottom deck compartment to assess the performance of the trailer in maintaining a satisfactory thermal and air quality environment for the pigs when loaded at full capacity and transported under cold and warm conditions. Because the upper and lower decks were separated by a floor/ceiling assembly with a rigid structural layer and sealed perimeter joints to minimize heat transfer between decks, and each deck operated with its own independent ventilation and environmental control system, air or heat exchange between levels was effectively prevented. As a result, the two decks were thermally isolated from one another, and loading pigs only in the bottom deck was sufficient to evaluate travel conditions encountered by the pigs.

**Figure 3:**
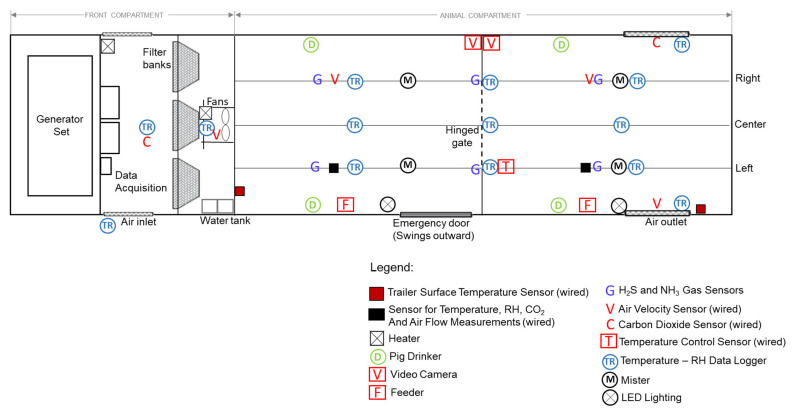
Diagram showing the arrangement and placement of components within the prototype trailer.

Thermal conditions and air quality in the animal compartment were monitored continuously throughout the duration of the trip to ensure that the minimum environmental requirements for pigs (according to the Code of Practice) inside the developed trailer were met and animal welfare was not compromised during transport. Temperature and RH in various locations in the prototype trailer were monitored using OM-EL-USB-TP-LCD and OM EL-USB-2 data loggers (Omega Engineering, Laval, QC, Canada; accuracy: ±0.5% (temperature) and ±3% (relative humidity)). The data loggers were spatially distributed at the top of each deck, approximately 1 m above the trailer floor, to avoid damage to sensors by animals. Additionally, real-time thermal conditions inside the trailer were monitored through thermistors and RH sensors connected to the mechanical ventilation control system. The relative humidity (%) recorded by data loggers was converted to humidity ratio (g/kg of dry air) to analyze the absolute moisture condition inside the animal compartment during transport [20]. Concentration of CO_2_ at four locations inside the trailer was measured using SE-0018 sensors (CO_2_Meter, Ormond Beach, FL, USA; range: 0 to 10,000 ppm; accuracy: ±3%). Hydrogen sulfide (H_2_S) and ammonia (NH_3_) level monitoring were performed using Dräger Pac 7000 (Draeger Safety Canada, Ltd., Mississauga, ON, Canada; range: 0–100 ppm (H_2_S), 0–300 ppm (NH_3_); detection limit: 1 ppm (H_2_S and NH_3_). Data for CO_2_ concentrations in the animal pens were logged continuously using a 16-port CR1000 data logger (Campbell Scientific, Edmonton, AB, Canada). All recorded data were downloaded and subsequently analyzed to achieve the objectives of the trial. The arrangement of sensors and other components within the trailer is shown in Figure 3.

Prior to each road test, body weight measurements from all pigs were carried out, and the physical condition and behavior of the pigs were visually assessed by trained animal care technicians to ensure that they were fit and healthy prior to being loaded into the trailer. Additionally, blood cortisol and rectal and body temperature were collected twice (before and after the trip) from the same pigs. During the 10-pig trips, all 10 pigs were sampled, whereas during the 40-pig trips, a random subset of 10 pigs was selected. All pigs had unique IDs, identified through ear notches, to ensure accurate identification. Rectal and body temperature were measured using a thermometer (Veterinary Electronic Thermometer, Xiyi Co. Ltd., Xi’an, China; accuracy: ±1 °C) and an infrared camera (C3-X, FLIR Systems Inc., Wilsonville, OR, USA; accuracy: ±3 °C), respectively. All handling procedures in our study were standardized, brief, and applied consistently across trips. Video cameras were installed in the animal compartment to monitor the behavior and overall welfare of the pigs during transport. Upon arrival at the destination, all pigs were visually inspected and samples were collected again to assess their physical condition and overall welfare.

### 2.3. Statistical Analysis

A SAS Mixed Model procedure with α = 0.05 (SAS 9.4, SAS Institute Inc., Cary, NC, USA) was performed to compare the mean differences of each environmental parameter (i.e., temperature, humidity ratio, CO_2_ levels, and air speed) across all monitoring locations inside the trailer. The model included trailer locations as a fixed factor and monitoring trips as a random factor. A test of normality was carried out using the Shapiro–Wilk test. If significant differences (*p* < 0.05) were observed between means, a Tukey or Tukey–Kramer post-hoc method was carried out to compare the means and consequently, to determine which pair of means was significantly different.

## 3. Results

The average values of temperatures, humidity ratio, and CO_2_ levels measured at different locations inside the trailer during the stable transport period from all 16 monitoring trips (n = 12 with 10 pigs and n = 4 with 40 pigs) are presented in the sections for stable temperature, humidity ratio, thermal comfort classification, and CO_2_ levels. The stable transport period typically starts about one hour after starting the trip and covers the longest duration in the transport process. This time period was chosen for the assessment of the overall trends in the environmental conditions in the trailer, after allowing time for the pigs to settle down from the disturbance during loading and for the operation of the installed ancillary systems to stabilize when the trailer attained highway travel speed with minimal interruptions due to traffic [21]. However, for the purpose of showing the variation in temperature, humidity ratio, and CO_2_ levels in the trailer during the entire transport period, only data from Trips 13 and 15 (warm conditions) and Trips 9 and 11 (cold conditions) are presented.

### 3.1. Temperature Level Distribution

#### 3.1.1. Temperature Time Series for the Entire Transport

**Figure 4:**
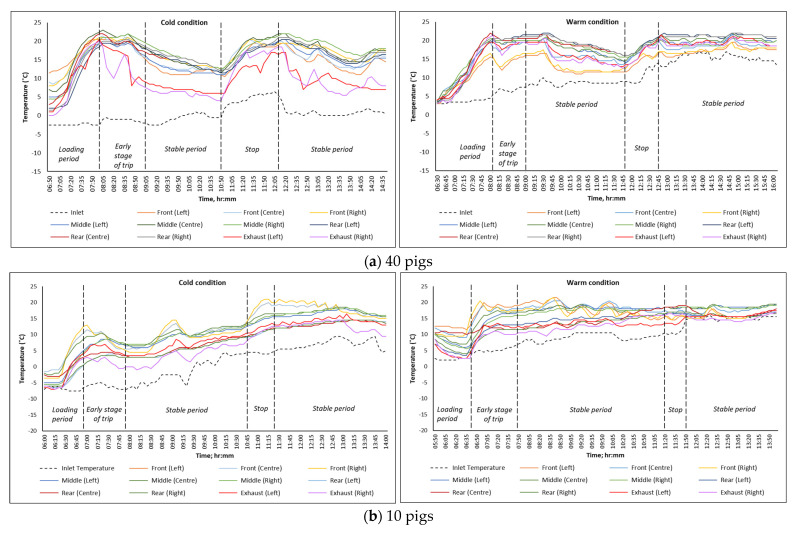
Variation in inlet, exhaust, and internal air temperature measured at different locations in the trailer when transporting (**a**) 40 pigs; (**b**) 10 pigs.

Figure 4 provides the spatial-temporal variation in temperature throughout the course of the monitoring trip to assess the thermal conditions inside the trailer when loaded at full capacity (40 pigs in the bottom deck) and partial capacity (10 pigs in the front compartment of the bottom deck) and transported during cold and warm weather conditions. Similar trends in temperature were observed for all monitoring trips in both warm and cold conditions. The temperature inside the animal compartment continually increased from the start of loading until it peaked during the early period of travel, which can be attributed to the heat production of the animals as a result of increased activities and adapting to the motion of the trailer during travel. This trend is consistent with the temperature variations observed in the other road tests when the trailer was partially loaded with 10 pigs only (shown in Figure 4b).

Under cold conditions, a decrease in temperature was observed during the early stage of the trip, see Figure 4a,b, when the inlet temperature was below 0 °C. However, the temperatures inside the animal compartment remained approximately 10 °C higher than the inlet temperature during this stage of the trip. The temperatures eventually increased and reached a maximum of 22 °C during the last four hours of the monitoring trip. For trips conducted under warm conditions, temperatures inside the animal compartment somewhat stabilized during the early stage of the transport period until the end of the trip, attaining average temperatures that ranged from 15 °C to 20 °C.

#### 3.1.2. Temperature During the Stable Transport Period

**Figure 5:**
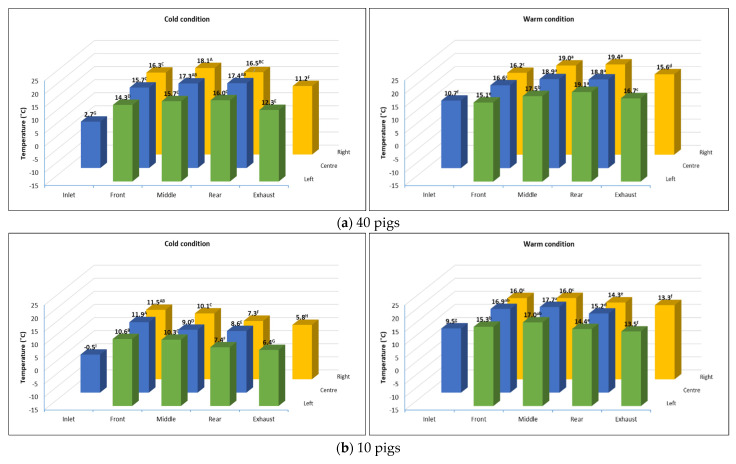
Average air temperature during the stable transport period at different monitoring locations in the trailer for (**a**) 4 trips loaded with 40 pigs during warm (n = 2) and cold (n = 2) conditions; (**b**) 12 trips loaded with 10 pigs during warm (n = 4) and cold (n = 8) conditions. Means with the same letters (uppercase for cold and lowercase for warm) are not significantly different (*p* > 0.05).

Figure 5 shows the average temperatures in the inlet, exhaust, and inside the animal compartment during the stable transport period over the course of all monitoring trips. The highest recorded temperatures were in the middle part of the trailer (between front and rear), where 80% of the pigs stayed, as seen from the videos from cameras installed inside the animal compartment. There was a gradual decrease in temperature from the front to the back part of the trailer for all trips, regardless of pig capacity and weather conditions. This is due to the three 900 W heaters attached to the rear grill of the axial fans, while exhaust was installed at the rear of the trailer. Under cold conditions, the average temperature in the animal compartment loaded with 40 pigs ranged from 14.3 °C to 18.1 °C, significantly higher (*p* < 0.05) than the average temperature obtained in trips loaded with only 10 pigs (9 °C to 11.9 °C). However, these temperatures for both 40-pig and 10-pig trips were 10 °C higher relative to the inlet temperature (about 0 °C), indicating that the heater effectively supplied warm air inside the animal compartment. Under warm conditions, the temperature trends were the same for 40-pig and 10-pig trips, with average temperatures inside the animal compartment ranging from 16 °C to 19.4 °C compared to the inlet temperature of 9.5 °C and 10.7 °C, respectively.

#### 3.1.3. Thermal Comfort Classification

**Figure 6:**
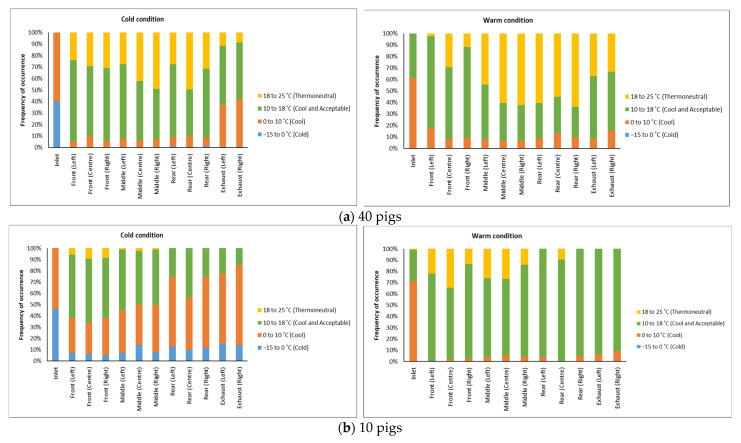
Thermal comfort classification at different monitoring locations in the trailer for (**a**) 4 trips loaded with 40 pigs during warm (n = 2) and cold (n = 2) conditions and (**b**) 12 trips loaded with 10 pigs during warm (n = 4) and cold (n = 8) conditions.

Thermal comfort classification is a concept used to categorize how comfortable animals are in their environment based on parameters like air temperature and humidity. This helps operators in maintaining animals within their thermoneutral zone. The thermal comfort classification, which was adapted from Xiong et al. [22], was categorized into four temperature ranges: cold (−15 °C < T < 0 °C), cool (0 °C < T < 10 °C), cool but acceptable (10 °C < T < 18 °C), and thermoneutral (18 °C < T < 25 °C). The thermal comfort classification for all monitoring trips, representing cold and warm transport conditions with loading 40 and 10 pigs, respectively, is shown in Figure 6. Generally, temperatures ranging from 10 °C to 18 °C occurred the most frequently in most trailer locations, with the middle section, where pigs were located most of the time, being generally warmer. It was also evident that temperatures in the animal compartment were higher during trips loaded with 40 pigs, regardless of weather conditions, compared to the trips loaded with 10 pigs. Temperatures inside the animal compartment for these trips were above 10 °C for 90% of the time, even though inlet temperatures of below 0 °C occurred 40% of the time under cold conditions, and inlet temperatures ranging from 0 °C to 10 °C occurred 60% of the time during warm conditions.

During transportation with 10 pigs only (shown in Figure 6b), the front compartment where the pigs were located maintained temperatures above 10 °C for 60% of the time, compared to below 10 °C and up to 45% occurrence of near-zero inlet temperature during the cold conditions. In contrast, during the warm conditions, the temperature inside the animal compartment remained consistently above 10 °C, while the inlet temperature ranged from 0 °C to 10 °C for 70% of the time.

### 3.2. Moisture Levels and Distribution

#### 3.2.1. Humidity Ratio for the Entire Transport

**Figure 7:**
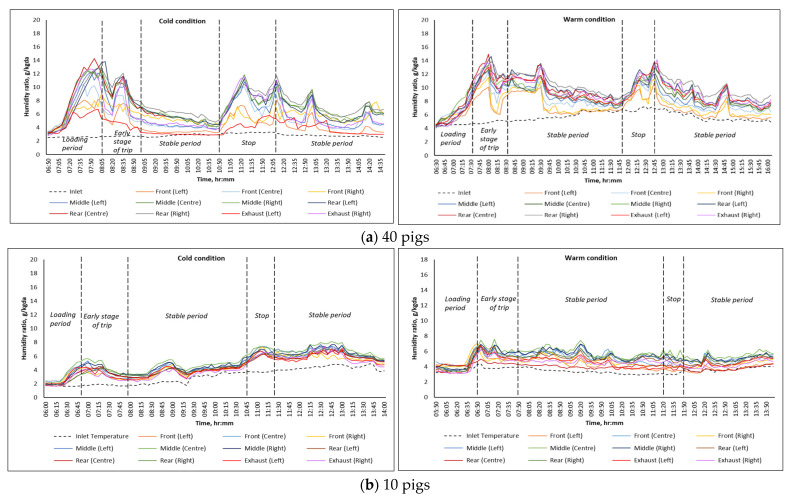
Variation in inlet, exhaust, and internal humidity ratio measured at different locations in the trailer when transporting (**a**) 40 pigs; (**b**) 10 pigs.

The humidity ratio accounts for both temperature and moisture content, and it is the most precise indicator to monitor heat and cold stress, air quality, and energy efficiency during animal transport. Figure 7 presents the humidity ratio at different locations of the trailer at different pig loading rates, representing cold and warm transport conditions, respectively. Generally, the humidity ratio in the trailer during both trips followed the same trend as the temperature series. The humidity ratio increased during the loading period, with an average highest peak of 14 g/kg dry air and 6 g/kg dry air for 40-pig and 10-pig trips, and then gradually decreased and stabilized as it reached the stable periods. The fluctuation in the humidity ratio under 40-pig trips was attributed to the periodic stops made during the trips, which were made to collect samples for monitoring air quality and animal welfare status. However, without these stops, it can be assumed that the trends for the trips loaded with 40 pigs would be comparable to those for trips with 10 pigs, as shown in Figure 7a,b.

#### 3.2.2. Average Humidity Ratio During the Stable Transport Period

**Figure 8:**
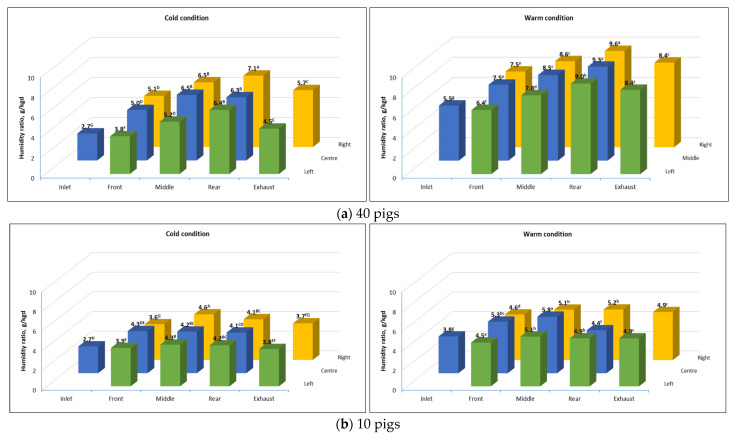
Average humidity ratio during the stable transport period at different monitoring locations in the trailer for (**a**) 4 trips loaded with 40 pigs during warm (n = 2) and cold (n = 2) conditions; (**b**) 12 trips loaded with 10 pigs during warm (n = 4) and cold (n = 8) conditions. Means with the same letters (uppercase for cold and lowercase for warm) are not significantly different (*p* > 0.05).

Figure 8 shows the average humidity ratios at different locations in the trailer during the stable transport period, with pigs transported at different capacities, under both cold and warm conditions. As expected, moisture levels inside the animal compartment during both transport conditions were significantly higher (*p* < 0.05) than the humidity ratios at the inlet. The highest average humidity ratio was measured in the middle of the animal compartment, where the pigs stayed, with higher levels of humidity ratio recorded on trips loaded with 40 pigs than on trips with 10 pigs. On trips with 40 pigs, the average humidity ratios were 6.3 g/kg of dry air in cold conditions and 8.8 g/kg of dry air in warm conditions, whereas on trips with 10 pigs, the average humidity ratios were 4.15 g/kg of dry air and 5.06 g/kg of dry air in cold and warm conditions, respectively. This increase in humidity ratio can be attributed to the levels of moisture released by the animals. Additionally, moisture levels at the exhaust were significantly higher (*p* < 0.05) compared to the inlet, which indicated that the exhaust air stream carried moisture with it.

### 3.3. Carbon Dioxide Levels and Distribution

#### 3.3.1. Carbon Dioxide Levels for the Entire Transport

**Figure 9:**
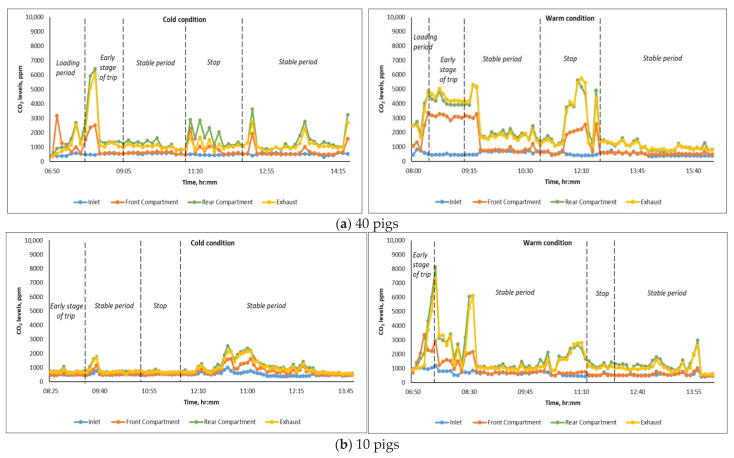
Variation in inlet, exhaust, and internal CO_2_ levels in the trailer when transporting (**a**) 40 pigs; (**b**) 10 pigs.

Carbon dioxide (CO_2_) levels were used as an indicator of overall air quality inside the trailer during transport. Figure 9 shows the CO_2_ concentration at the inlet, exhaust, and inside the animal compartment during the 40-pig and 10-pig trips under cold and warm transport conditions. Over the course of the monitoring trips, CO_2_ levels inside the animal compartment generally followed the same trend as temperature and humidity ratio. Peaks in CO_2_ levels, like temperature and moisture levels, can be attributed to the pigs’ increased physical activity in the animal compartment during loading, slowdowns, and travel interruptions, during which pigs tend to become agitated. Subsequently, CO_2_ levels usually stabilized a few minutes after loading and after resuming travel, following short interruptions.

#### 3.3.2. Carbon Dioxide Levels During the Stable Transport Period

**Figure 10:**
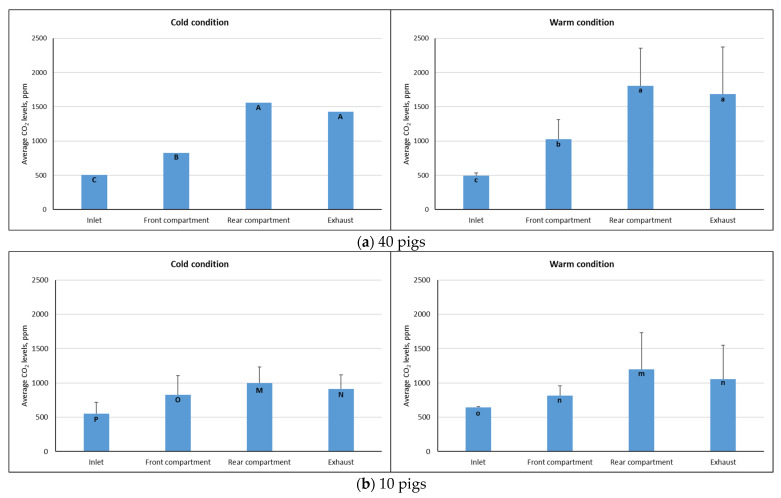
Average CO_2_ levels during the stable transport period at different monitoring locations in the trailer for (**a**) 3 trips loaded with 40 pigs during cold (n = 1) and warm (n = 2) conditions; (**b**) 11 trips loaded with 10 pigs during cold (n = 8) and warm (n = 3) conditions. Means with the same letters (uppercase for cold and lowercase for warm) are not significantly different (*p* > 0.05).

Figure 10 shows the average CO_2_ levels during the stable transport period at different monitoring locations in the trailer during the monitoring trips under cold and warm conditions. Consistent with the average temperature and humidity ratio, CO_2_ levels recorded in 40-pig trips were higher than the carbon dioxide levels obtained in the 10-pig trips, regardless of weather conditions. During cold conditions, the average CO_2_ levels inside the animal compartment ranged from 827 ppm to 1558 ppm for 40-pig trips and from 825 ppm to 1000 ppm for 10-pig trips. These values were lower than the CO_2_ levels observed in warm conditions, which ranged from 1025 ppm to 1803 ppm and 817 ppm to 1198 ppm for trips loaded with 40 and 10 pigs, respectively. The highest CO_2_ concentrations were observed in the rear and exhaust vents under both cold and warm conditions, indicating that the ventilation system was effective in removing stale air from inside the trailer (shown in Figure 10).

### 3.4. Air Speed

**Figure 11:**
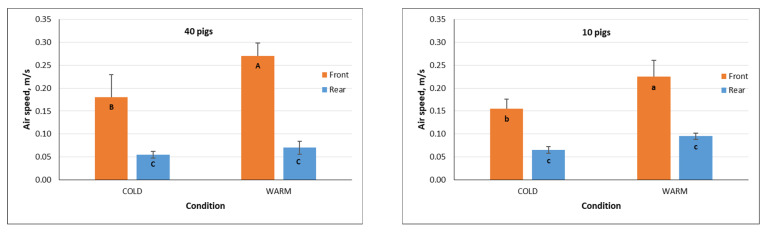
Average air speeds during the stable transport period at the front and rear locations in the trailer for 4 trips loaded with 40 pigs during cold (n = 2) and warm (n = 2) conditions and 4 trips loaded with 10 pigs during cold (n = 2) and warm (n = 2) conditions. Means with the same letters (uppercase for cold and lowercase for warm) are not significantly different (*p* > 0.05).

Figure 11 shows the average air speeds during the stable transport period at the front and rear locations of the trailer when loaded with 40 and 10 pigs under cold and warm transport conditions. The average air speeds in the front compartment were significantly higher (*p* < 0.05) than those in the rear compartment across all transport conditions and loading densities. This difference is attributed to the proximity of the front compartment to the fan, which generated more turbulent flow. For the 40-pig trips, the average air speeds inside the animal compartment ranged from 0.06 to 0.18 m/s during cold conditions and 0.07 to 0.27 m/s during warm conditions. For the 10-pig trips, the average air speeds ranged from 0.07 to 0.16 m/s and 0.10 to 0.23 m/s during cold and warm conditions, respectively. These observed values are within the recommended 0.25 m/s air speed set by the National Farm Animal Care Council for protection of nursery pigs from drafts [23].

In addition, air speeds were consistently higher during warm transport conditions than during cold conditions, with the difference most pronounced in the front compartment. This was expected given the higher ventilation rates required during warm weather. During warm conditions, the average ventilation rates were 20,838.7 m^3^/h for 40-pig trips and 14,368 m^3^/h for 10-pig trips. During cold conditions, the average ventilation rates were 10,609.2 m^3^/h and 9438.5 m^3^/h for 40 and 10-pig trips, respectively.

### 3.5. Animal Welfare

Table 1 summarizes the average body and rectal temperatures of pigs, as well as their blood cortisol levels during transport. Rectal temperature measures the core body temperature and can be used to evaluate thermal comfort and heat/cold stress, while blood cortisol reflects the pigs’ overall response to stress [24]. Blood cortisol levels vary at different monitoring trips but are deemed insignificant (*p* > 0.05), while rectal temperatures were considerably stable (ranging from 38.1 °C to 39.8 °C) from the start to the end of the monitoring trips. Rectal temperatures were comparable to the normal rectal temperature of 38.4 °C to 39.8 °C [25], which indicates that pigs remained within their normal thermoneutral zone during transport. In contrast, body temperature shows slight fluctuations, possibly due to varying heat distribution and heat buildup during transport. Overall, there was no significant change in the levels of blood cortisol and the rectal and body temperatures of pigs measured at the start and end of each monitoring trip. These results suggest that the pigs in the trailer did not experience any stress during transport. This is evident from the videos taken during the trips, which showed no signs of bunching, and no dead pigs were recorded over the course of 16 monitoring trips.

## 4. Discussion

### 4.1. Temperature Distribution and Thermal Comfort Classification

Temperatures at different locations within the trailer were recorded to evaluate the effectiveness of the heating system relative to the inlet temperature and to better understand airflow distribution in the compartments loaded with pigs. As shown in Figure 4, there was no noticeable difference in the temperature trends between trips loaded to full capacity (40 pigs) and trips loaded with only 10 pigs. However, there was a noticeable increase in temperature from the start of loading and during the early stage of travel in most of the monitoring trips. This observation aligns with previous studies, which reported a rapid temperature rise within 45 min of loading and 30 min of travel before the temperature stabilized [6,26]. This increase in temperature is primarily attributed to increased activity during loading and handling. Pig behavior was also monitored using installed cameras, revealing that the animals stood closely together during the early stages of the trips and remained lying down for most of the trips. This standing behavior helps the pigs adapt to motion while also attempting to reduce conductive heat loss to the flooring, especially during cold transport conditions [27].

The stable transport period, which covered the longest interval during the transport process, was selected to assess the general overall trends in the resultant thermal conditions in the trailer. In cold conditions, the average temperature in the animal compartment with 40 pigs ranged from 14.3 °C to 18.1 °C, significantly higher (*p* < 0.05) than the 9 °C to 11.9 °C in trips with only 10 pigs. The temperature difference may be attributed to the difference in the number of pigs, as it directly affects the body heat generated throughout the trips. Despite this, the achieved temperatures were still 10 °C higher than the near-zero inlet temperature (−0.5 °C). Additionally, these temperatures in the animal compartment during the cold conditions were higher compared to the results from previous studies using conventional trailers. Brown et al. [6] found that the average temperature in the animal compartment of conventional (potbelly) transport trailers in the winter ranged from −7.75 °C to 5.59 °C. Other studies recorded a much lower animal compartment temperature, with an average of −4 °C [26] to −10 °C [13], which is deemed detrimental to pigs, causing severe stress that resulted in an increase in DOA or non-ambulatory cases during transport. In contrast, the average temperature inside the animal compartment obtained during the warm condition ranged from 16 °C to 19.4 °C, which falls within the 5 °C to 30 °C temperature range recommended by the European Union Council Regulation 1/2005 for animal transport vehicles [28]. This is comparable to the studies conducted by Brown et al. [6] using conventional potbelly trailers, with temperatures ranging from 15.34 °C to 18.25 °C during summer periods.

In addition, the temperatures inside the animal compartment, regardless of pig loading and weather conditions, followed a decreasing trend from front to back, with temperatures at locations close to the fans significantly higher (*p* < 0.05) than at locations near the exhaust. This trend is due to the placement of the heating system and ventilation system configuration in the trailer, which resulted in a general front-to-rear air flow pattern inside the animal compartment. Heaters were installed in the fans located at the front end of the trailer, while exhaust openings are located at the rear. This is in contrast to the reported rear-to-front air movement inside the conventional livestock transport trailer due to the differences in external pressure fields while the vehicle is in forward motion [6,14,15]. This caused the temperature and other environmental factors (like moisture and CO_2_ levels) in conventional trailers to generally increase from the rear to the front. However, a study by Xiong [29] that characterized the thermal environment during 43 farm-to-abattoir transport of pigs under various weather conditions found a different air flow pattern, which showed to be coming from the middle of the conventional trailer to either the front or rear of the vehicle.

Over the course of 16 monitoring trips conducted in this study, temperatures between 10 °C and 18 °C were achieved across trailer locations. When transporting 40 pigs, the animal compartment remained above 10 °C for over 90% of the time, despite 40% of inlet temperatures below 0 °C during cold conditions, and 60% of below 10 °C inlet temperatures during warm conditions. The temperatures achieved in this study fall within the recommended thermal comfort zone, ranging from 10 °C to 24 °C, which was set by the National Farm Animal Care Council in Canada [23]. For trips with 10 pigs, the front compartment where the pigs were located maintained temperatures above 10 °C for 60% to 98% of the time during both cold and warm conditions. Notably, with 40% of near-zero inlet temperatures during cold conditions, the temperatures in trips loaded with 10 pigs were colder compared to trips loaded with 40 pigs. This suggests that the body heat generated by the pigs played a significant role in raising the environmental temperature inside the animal compartment. Nonetheless, the heating system was able to increase the temperature significantly, despite the severe ambient temperature and a smaller number of pigs. Furthermore, the maximum temperature and relative humidity recorded during warm conditions were 23 °C and 90%, respectively, which are still within the thermoneutral zone (outside of the alert zone) based on the temperature and humidity stress index for transporting pigs developed by the National Farm Animal Care Council in Canada [23].

It is critical to keep the temperature in the trailer above 0 °C for most of the time because prolonged exposure to cold or extreme cold conditions can deplete energy reserves of the animals and compromise their overall welfare and meat quality [30]. In reality, pigs were inevitably transported under extreme weather conditions, resulting in temperature fluctuation and failure to achieve the recommended thermal comfort zone [1,17]. In addition, uneven temperature distribution within different compartments was observed in potbelly trailers, resulting in poor ventilation and therefore inducing additional stress to pigs [5,22]. These issues were mitigated by using a mechanically ventilated trailer to maintain an acceptable temperature during extreme environmental conditions.

### 4.2. Moisture Levels and Distribution

In addition to temperature, regulating humidity levels during animal transport is crucial for ensuring animal welfare. Although no specific humidity thresholds are established, studies have observed that RH inside transport trailers can vary significantly depending on bedding levels, trailer design, and ambient conditions, which contribute to animal stress [17]. The higher humidity ratio recorded in the animal compartment could be attributed to the moisture from animals, such as waste, urine, animal respiration, and drinking water wastage. Maintaining a suitable ventilation flow rate and allowing fresh air inside the animal compartment is essential to keep an acceptable moisture level across all locations in the trailer.

High moisture levels in the animal compartment are not deemed detrimental during winter conditions because the temperatures in these locations were generally above freezing; thus, the possibility of frostbite is minimized. On average, humidity ratios in the animal compartment ranged from 4.15 to 6.3 g/kg dry air during cold transport conditions, slightly higher than the mean humidity ratio of 1.8 to 4.9 g/kg of dry air observed by Brown et al. [6] in conventional pig transport trailers during winter periods. On the other hand, humidity ratio levels in the animal compartment during warm weather ranged from 5.06 to 8.8 g/kg dry air, consistent with the 6.8 to 7 g/kg dry air reported by Weschenfelder et al. [5] in potbelly trailers. Although this study used an enclosed trailer, it is still crucial to provide sufficient fresh air during winter periods and ensure that the exhaust airstream effectively removes moisture from the air to maintain acceptable moisture levels inside the animal compartment during transport.

### 4.3. Carbon Dioxide Levels and Distribution

As shown in Figure 9, CO_2_ levels during cold and warm transport periods significantly increased (*p* < 0.05) as the airstream moved from the inlet to the rear compartment. In addition, CO_2_ levels at the exhaust were significantly higher (*p* < 0.05) than the inlet CO_2_ levels, indicating that the ventilation system was able to remove stale air from inside the trailer under the specified operating conditions. On average, CO_2_ concentration inside the animal compartment during transport under cold conditions was 1192.70 ppm and 912.23 ppm for trips with 40-pig and 10-pig capacity, respectively. These levels are relatively lower than the CO_2_ concentration of 1424.5 ppm reported by Cabahug [21] using the same enclosed trailer, but in an unmodified condition, as well as CO_2_ levels recorded in pig gestation rooms (1900–2300 ppm) during the cold season [31]. On the other hand, under warm conditions, CO_2_ levels in the animal compartment were 1007.82 ppm for trips loaded with 10 pigs and 1414.19 ppm for 40-pig trips; these levels were comparable to the reported mean CO_2_ concentrations in existing commercial livestock vehicles (878–2746 ppm) running at summer ventilation rates [15].

Throughout the 16 monitoring trips, measurements of hydrogen sulphide (H_2_S) and ammonia (NH_3_) produced no readings that are above the detection limit of the sensors (1 ppm for NH_3_ and H_2_S). These gases are not expected to be a concern in pig transport because the animals are only held in the transport container for a short period of time, which is not sufficient to produce measurable levels of the gases under normal transport conditions.

### 4.4. Animal Welfare

Body temperature and blood cortisol levels are used in the assessment of pigs’ response to transport stress [24,32]. Table 1 shows the summary of average blood cortisol levels, as well as rectal temperatures and body temperatures from 4 monitoring trips using 40 pigs. Although the difference in average blood cortisol levels measured from the start and end of the trip was deemed insignificant (*p* > 0.05), it can be observed that the cortisol levels in Trip 1 and Trip 3 decreased further at the end of the trip, while the slightly increased levels at the end of Trip 2 were deemed negligible. The varying cortisol levels at the start of all monitoring trips may be attributed to environmental conditions during loading. The ambient temperature during loading in Trip 3 was colder, ranging from −2.5 °C to −1 °C, compared to Trip 1 and Trip 2, where the temperatures were about 5 °C to 7 °C. Furthermore, the cortisol levels observed in this study (72–148 nmol/L) were considerably lower than those reported by Warriss et al. [33], which ranged from 147.9 nmol/L to 159.5 nmol/L in pigs transported using natural and fan-assisted ventilation systems. Rectal temperatures remained relatively stable, ranging from 38.2 °C to 39.8 °C with an average of 39.3 °C. This is comparable to the normal rectal temperatures of nursery pigs [25] and slightly higher than the reported average gastrointestinal tract temperature (GTT) of 38.8 °C under winter and summer transport conditions [34]. On the other hand, body temperatures show slight fluctuations, which is influenced by varying environmental conditions, heat distribution, and heat buildup during transport.

Overall, there was no significant change (*p* > 0.05) in the pigs’ blood cortisol levels, rectal temperatures, or body temperatures from the start to the end of each monitoring trip. This suggests that the pigs showed reduced or minimal stress during transport.

## 5. Conclusions

The findings of this study demonstrated that the ancillary systems (e.g., ventilation and heating systems) installed in the trailer were capable of maintaining acceptable air quality and thermal environment for the pigs during transport under cold and warm weather conditions. On average, the temperatures in the animal compartment occupied by pigs were above 10 °C despite the near-zero temperature in the trailer air inlet. The temperature in the middle section of the trailer, where pigs stayed most of the time, frequently experienced extended periods of cool but acceptable temperature (10 °C to 18 °C). Moisture levels and CO_2_ concentration in the animal compartment were comparable to the humidity ratios and CO_2_ levels observed in conventional pig transport trailers during winter periods. Furthermore, pigs showed reduced or minimal stress, and no dead pigs were recorded throughout the 16 monitoring trips. These findings highlight the significant potential of this trailer design to mitigate thermal stress and environmental discomfort, thereby promoting animal welfare and reducing the risks of transport-related morbidity and mortality. Further testing of the trailer under a wider range of environmental conditions, particularly during peak summer conditions and with pigs from different production stages (i.e., weaning, finishing), is recommended to fully characterize its overall transport performance.

## Figures and Tables

**Table 1:** Pig temperature and blood cortisol levels during transport.

Monitoring Trips	Blood Cortisol, nmol/L		Rectal Temperature, °C		Body Temperature, °C	
	Start	End	Start	End	Start	End
1	82.3 ± 40.2	72.0 ± 21.6	39.4 ± 0.5	38.1 ± 0.7	32.9 ± 1.4	35.1 ± 0.7
2	109.0 ± 57.5	114.8 ± 57.5	38.9 ± 0.3	39.2 ± 0.4	33.4 ± 0.8	34.0 ± 0.9
3	148.0 ± 35.0	81.7 ± 40.6	39.7 ± 0.4	39.6 ± 0.3	34.1 ± 1.4	39.6 ± 0.2
4	-	-	39.8 ± 0.3	39.6 ± 0.3	32.0 ± 1.2	33.5 ± 1.7

## Data Availability

The original contributions presented in this study are included in the article. Further inquiries can be directed to the corresponding author.

## Abbreviations

The following abbreviations are used in this manuscript:

CO_2_	Carbon dioxide
DOA	Dead on arrival
H_2_S	Hydrogen sulfide
NH_3_	Ammonia
RH	Relative humidity
